# Supplementary material for: Gravitational-wave asteroseismology with fundamental modes from compact binary inspirals
Source: Nat Commun. 2020 May 21;11:2553. doi: 10.1038/s41467-020-15984-5 (PMC7242351; doi:10.1038/s41467-020-15984-5)
Supplement: Supplementary file 1 — Supplementary Information [file 41467_2020_15984_MOESM1_ESM.pdf]

Supplementary Information to Pratten et al: Gravitational-Wave Asteroseismology with Fundamental Modes from Compact Binary Inspirals

Geraint Pratten,[1,2,*] Patricia Schmidt,[1] and Tanja Hinderer[3,4]

[1] *School of Physics and Astronomy and Institute for Gravitational Wave Astronomy, University of Birmingham, Edgbaston, Birmingham, B15 9TT, United Kingdom*
[2] *Universitat de les Illes Balears, Crta. Valldemossa km 7.5, E-07122, Palma, Spain*
[3] *GRAPPA, Anton Pannekoek Institute for Astronomy and Institute of High-Energy Physics, University of Amsterdam, Science Park 904, 1098 XH Amsterdam, The Netherlands*
[4] *Delta Institute for Theoretical Physics, Science Park 904, 1090 GL Amsterdam, The Netherlands*

(Dated: April 6, 2020)

Supplementary information to Gravitational-Wave Asteroseismology with Fundamental Modes from Compact Binary Inspirals. This document contains 3 Figures and 1 Table.

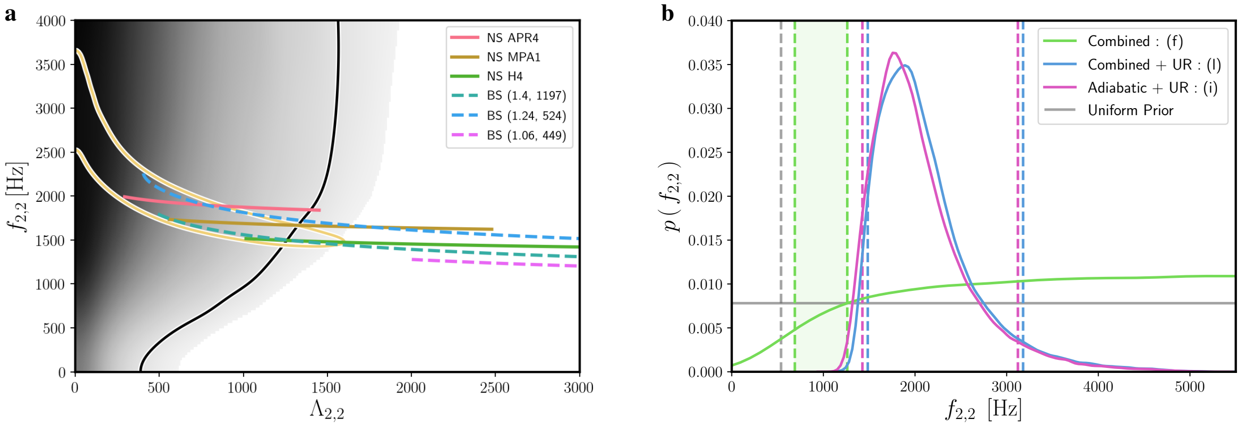

Supplementary Figure 1. **Complementary to Fig. 1 in the main paper, showing the results for the smaller-mass object m_2 of GW170817**. **a** Two-dimensional PDF for the f_2-mode frequency and tidal deformability. The solid lines correspond to the 90% credible regions, where the black curve corresponds to the analysis in which $f_{2,\mathrm{A}}$ is treated as an independent parameter and the yellow one to imposing the universal relations, i.e. fixing $f_{2,\mathrm{A}}$ given $\Lambda_{2,\mathrm{A}}$. The posteriors are overlaid with UR predictions for three EoS for NSs (coloured solid curves), and three massive BSs (coloured dashed curves) denoted $(m_\mathrm{b}/m_\mathrm{n}, \lambda_\mathrm{b})$, with $m_\mathrm{n} = 1.675 \times 10^{-27}$kg being the neutron mass, where all curves are restricted to the 90% interval of the component mass posterior, $m_2 \in [1.04, 1.37]\ \mathrm{M}_\odot$. **b** Marginalised one-dimensional PDF for the f_2-mode frequency. We show results for the following three tidal phase models as listed in Tab. I of the main paper: (i) purely adiabatic tides with URs imposed (pink), (l) adiabatic and dynamical tides with UR imposed (blue) and (f) adiabatic and dynamical tides without UR assumed (green). The dashed lines indicate the corresponding 90% lower bound (green) or CI (pink and blue). The shaded region indicates the range of the lower bound under a varying upper prior limit on $\Omega_{2\mathrm{A}}$.

Network	SNR
HLV	71
3 A+	145
3 HF4S	170
ET-D	788
CE	1107
ET + 2 CE	1753

Supplementary Table I. **Network SNRs of a GW170817-like source at 40 Mpc.** Here we show the network SNRs for all detector configurations considered in Fig. 2 of the main paper.

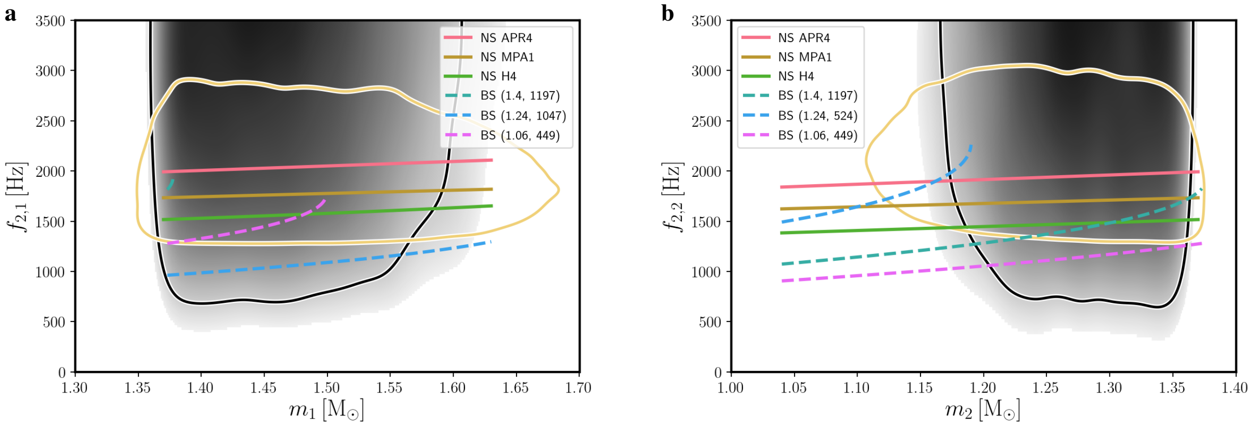

Supplementary Figure 2. **Joint two-dimensional PDF for component mass and quadrupolar f-mode frequency for GW170817**. The information displayed here complements Fig. 1 in the main text by showing explicitly the information on the masses from our analysis. Providing f-mode and mass information without reference to the tidal deformability is useful for direct constraints as most calculations of compact-object oscillation modes are done within a quasi-normal modes framework that only gives the frequency but not Λ_ℓ. **a** Two-dimensional PDF for the f_2-mode frequency and the mass of the larger companion m_1. The solid lines represent the 90% credible region, where the black-shaded region denotes the analysis in which $f_{2,A}$ is treated as an independent parameter and the yellow curve to imposing the universal relations. The posteriors are overlayed with UR predictions for three EoS for NSs (the solid coloured curves) and three massive BSs (coloured dashed curves). **b** Two-dimensional PDF for the f_2-mode frequency and the mass of the smaller companion m_2. The shaded regions as described for panel **a**.

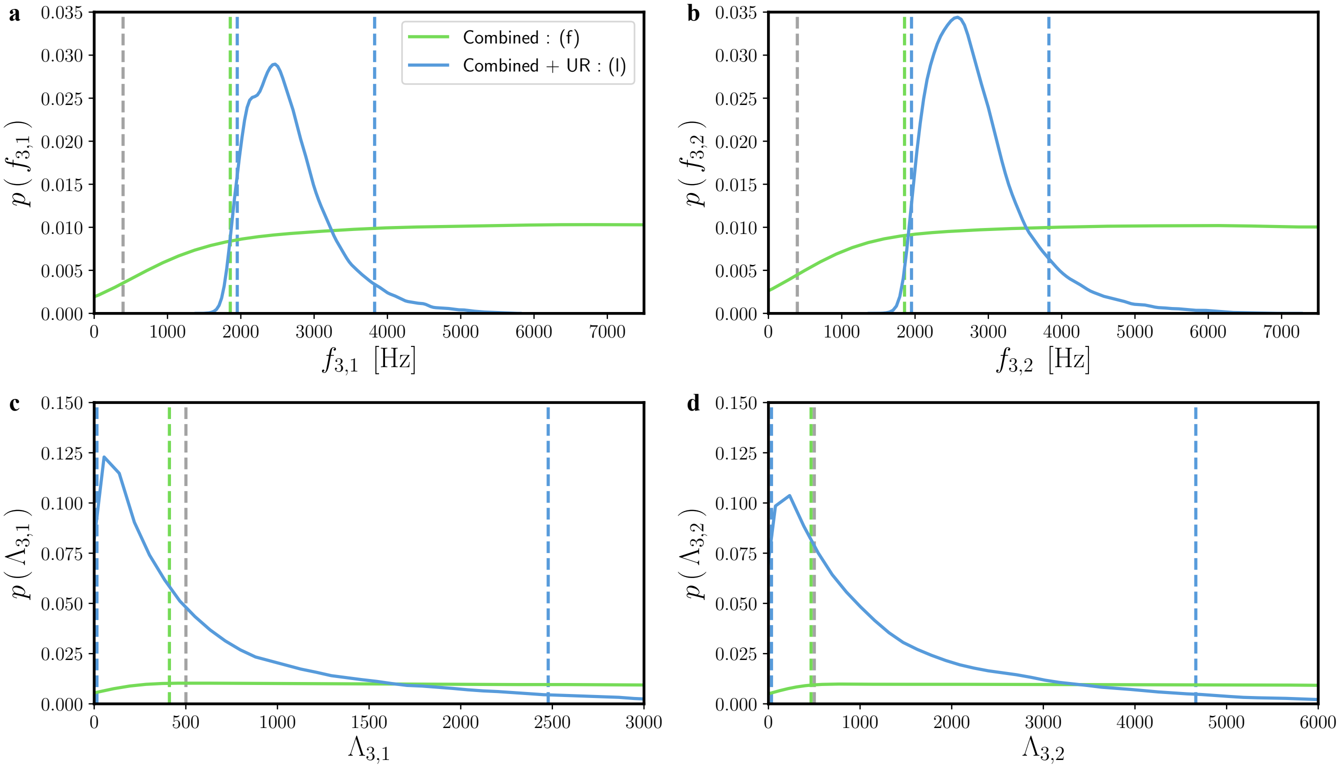

Supplementary Figure 3. **Constraints on the octupolar f-mode frequency and octupolar tidal deformability for GW170817**. Note that for the unconstrained tidal deformabilities, our results are manifestly dominated by the prior with the posteriors demonstrating a slight shift towards smaller values of Λ_3, as predicted by the URs. As with the quadrupolar f-modes, the lower bound on f_3 is in agreement with the lower limit implied by imposing URs. **a** Octupolar f-mode frequency for the larger companion in GW170817. **b** Octupolar f-mode frequency for the smaller companion in GW170817. **c** Octupolar tidal deformability for the larger companion in GW170817. **d** Octupolar tidal deformability for the smaller companion in GW170817.
